# Primary Kaposi’s sarcoma of the heart in non-immunodeficient patient: case report and literature review

**DOI:** 10.1186/s13000-015-0354-5

**Published:** 2015-07-19

**Authors:** Eka Makharoblidze, Nana Goishvili, Maia Mchedlishvili, Mikheil Jangavadze

**Affiliations:** Department of Pathology, Aleqsandre Natishvili Institute of Morphology, TSU, 2 Chiaureli Str, Tbilsi, 0159 Georgia

**Keywords:** Kaposi’s sarcoma, Heart, Immunohistochemistry

## Abstract

We present a case of Kaposi’s sarcoma (KS) of the heart in a 45-year-old non-immunodeficient woman with symptoms of pericardial effusion and cardiac tamponade. Computed Tomography (CT) coronary angiography and transesophageal echocardiography (TEE) showed a low-density tumorous mass (50 mm diameter) at the level of auricle of the right atrium spreading towards the superior vena cava, floating in the cavity of the right atrium. On histological examination, the tumor consisted of fibrovascular connective tissue with areas of necrosis and hemorrhage. Fibrous septae contained different sized thin walled capillary type blood vessels and lymphangioma-type vascular spaces. Vascular spaces were surrounded by extravasated erythrocytes, deposits of hemosiderin and sparse lymphoplasmacytoid infiltrates. On the periphery of tissue fragments and around vascular spaces, there was a cellular kaposiform proliferation of the spindled cells. Slit-like spaces between spindle cells contained erythrocytes. Nuclear pleomorphism of the spindled cells was minimal. Few mitotic figures were present. Spindle cells were Vimentin, CD34 and CD31 positive. More than 10 % of spindled cells were Ki67 positive. This characteristic histology and immunohistochemistry is consistent with Kaposi’s sarcoma. Patient has no history of other malignancies and no other primary tumor was detected. Patient also was negative for HIV infection. There are only 10 documented cases of primary Kaposi’s sarcoma of the heart in non-immunodeficient patients reported in the current medical literature. Our report is the first case in which imaging, histology and immunohistochemistry data are available.

**Electronic supplementary material:**

The online version of this article (doi:10.1186/s13000-015-0354-5) contains supplementary material, which is available to authorized users.

## Background

Primary tumors of the heart are very rare. Lifetime incidence ranges between 0.0017 to 0.02 % [1]. Only about 6 % of these tumors are malignant [2]. One third of them are angiosarcoma, but other malignant vascular tumors such as Kaposi’s sarcoma and malignant epithelioid hemangioendothelioma are extremely rare. There are only 10 reported cases of primary cardiac Kaposi’s sarcoma in non-immunodeficient persons in literature [3–11].

**Table 1:** Summaries of the 11 cases (including original) of the Primary Kaposi’s sarcoma of the heart

Case no.	Author	Year	Age	Sex	Site
1	Choisser, R. M. et. al. [5]	1939	26	Male	Right atrium
2	Choisser, R. M. et. al. [5]	1939	30	Male	Right atrium
3	Da Cunha Motta, L. [10]	1941	46	Male	Right atrium, pericardium
4	Aegerter, E. et al. [8]	1942	60	Male	Right atrium
5	Contreras, R. [6]	1957	43	Male	Right atrium
6	Gelfand, M. [7]	1957	59	Male	Right atrium
7	Ayer, J. P., et al. [9]	1962	38	Female	Left atrium
8	Rozegnal, W., et al. [3]	1973	Adult	Male	-
9	Levison, D. A. et al. [4]	1976	14	Male	Right atrium
10	Noohi, F., et al. [11]	1997	58	Male	Multiple lesion of the heart and pericardium
11	Original case	2013	45	Female	Right atrium, pericardium

Kaposi’s sarcoma (KS), which was described by Hungarian dermatologist Moritz Kaposi in 1872 [12], occurs mainly in skin, but visceral organs also get affected, especially in patients with AIDS [13, 14]. Nowadays, it is suggested, that KS is associated with superinfection by human herpesvirus 8 (HHV8, also known as Kaposi’s sarcoma [KS]-associated herpesvirus) in immunodeficient patients [15–17]. However, in immunocompromised patients, subepicardial adipose tissue is more commonly involved than the myocardium or endocardium, compared to sporadic cases [18, 19].

Cardiac KS lesion mostly remains unrecognized during clinical and imaging investigation and is diagnosed only by pathologists. It is suspected that, before introduction of molecular techniques, such lesions have been confused with primary angiosarcoma of the heart, which is more common, than Kaposi’s sarcoma [4]. Moreover, some authors suggest [20], that early reports of unusual cases of primary heart fibrosarcomas also can be classified as a Kaposis’s sarcoma [21].

Here we present the case of a 45 year old non-immunodeficient woman with primary Kaposi’s sarcoma of the heart and a review of 10 cases, which we found in the medical literature through PubMed search using the search terms “Kaposi’s sarcoma” AND “heart”.

## Case presentation

### Clinical history and radiologic findings

**Fig. 1:**
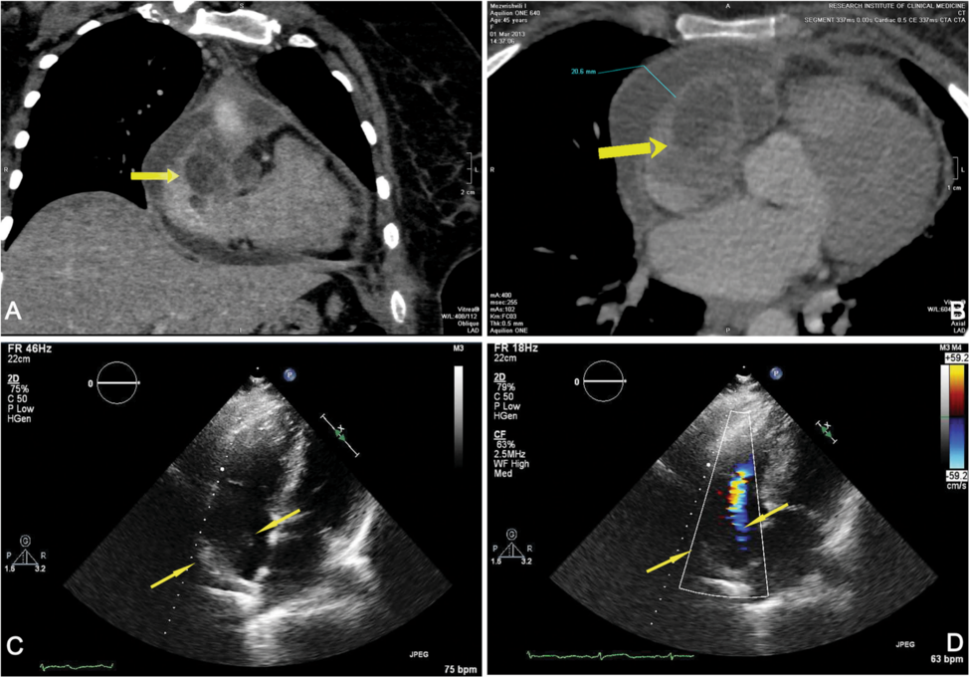
**a**, **b** Computed Tomographic (CT) coronary angiography. Low-density tumorous mass (50 mm in diameter) at the level of auricle of the right atrium. **c**, **d** Transesophageal echocardiography. Tumor in the auricle of the right atrium, spreading toward superior vena cava

45 year old female patient with symptoms of pericardial effusion and cardiac tamponade was referred to the clinic. Pericardiothentesis was performed immediately. Microbiological and cytological investigation of the pericardial fluid showed no evidence of an infection or malignancy. On the Computed Tomographic (CT) coronary angiography at the level of auricle of the right atrium, low-density tumorous mass (50 mm in diameter) was found (Fig. 1a, b) (An additional movie file shows this in more detail [see Additional file 1]). No other abnormalities of cardiac structures were identified. TEE showed a tumor in the auricle of the right atrium extending towards superior vena cava. Free end of the tumor was floating in the cavity of right atrium (Fig. 1c, d) (Additional movie files show this in more detail [see Additional file 2 and Additional file 3]). Surgery was done 3 days after admission to the hospital. Non-complete surgical removal of the tumor was performed due to anatomic features of the tumor growth. The patient recovered and was discharged 9 days after the operation. On 10 month follow-up, the patient remained asymptomatic.

Paraffin embedded tissue samples from the pericardium and resected intracardiac tumor (auricle of the right atrium) were sent to our laboratory for reference.

### Histopathologic findings

**Fig. 2:**
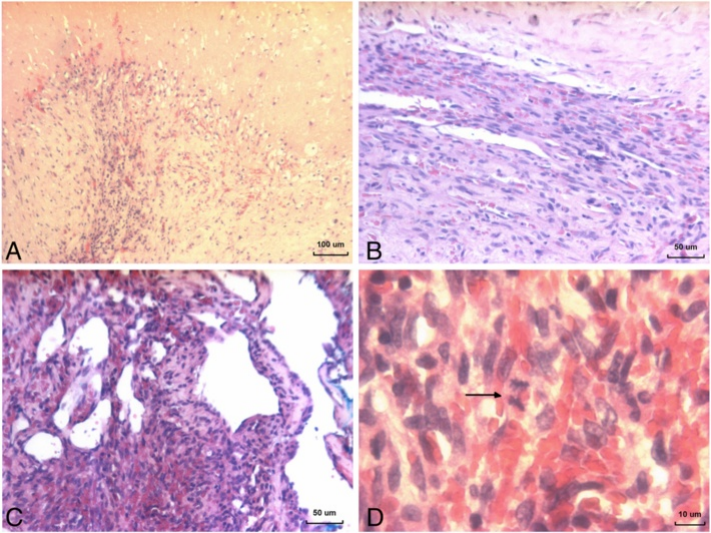
**a** Areas of necrosis and hemorrhage surrounded by connective tissue septa. H&E; (**b**) Kaposiform proliferation of the spindled cells with slit-like spaces. H&E; (**c**) Slit-like spaces contains erythrocyte. H&E; (**d**) Spindled cell with mitotic figure (*arrow*). H&E

Tissue fragments had heterogeneous structures. One sample consisted of fibrovascular connective tissue with adipose tissue inclusions. Capillary and venous type blood vessels, with perivascular lymphoplasmocytoid infiltration were found in fibrose tissue. Two other tissue fragments generally were characterized by erythrocytes and “ghost” cells. Areas of necrosis and hemorrhage were surrounded by connective tissue septa (Fig. 2a). They contained variable-sized, thin-walled capillary-type blood vessels and lymphangioma-type vascular spaces, which were embedded in swollen collagen and hyaline matrix. There was a cellular kaposiform proliferation of the spindled cells present focally, on the periphery and around vascular spaces. Slit-like spaces between spindle cells contained erythrocytes (Fig. 2b, c). In such areas, there were numerous extravasated erythrocytes, haemosiderophages and plasma cells. Nuclear pleomorphism of the spindled cells was minimal. Few mitotic figures were present (Fig. 2d).

**Fig. 3:**
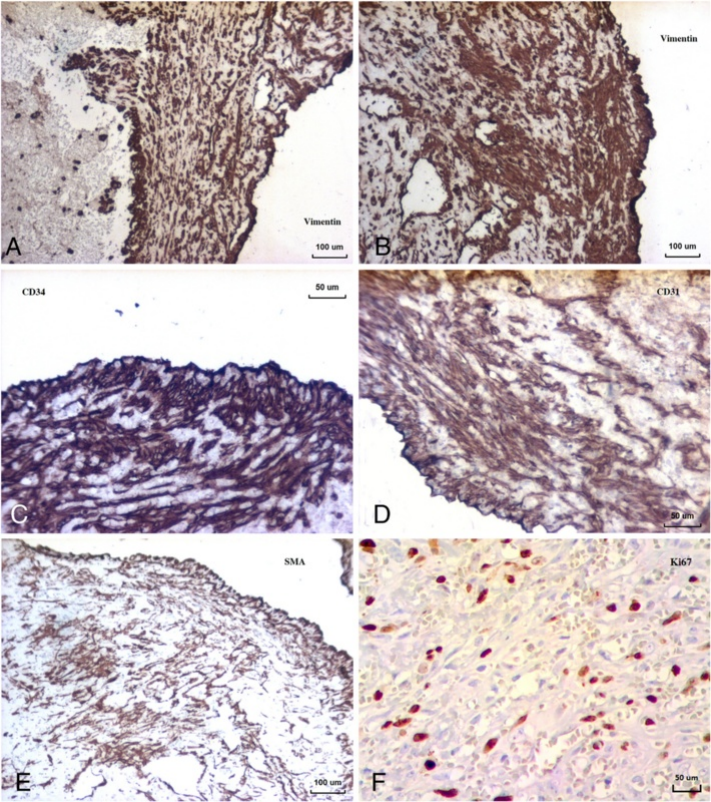
Immunohistochemistry: (**a**, **b**) Spindled cells with positive cytoplasmic immunostaining for vimentin; (**c**) Tumor cells express CD34 antigen; (**d**) Tumor cells also positive for CD31; (**e**) Some cells (pericytes) with positive cytoplasmic immunostaining for αSMA and (**f**) About 10 % of spindled cells express Ki67 (Mib1) antigen

Spindle cells were positive for vimentin, CD34 and CD31 (Fig. 3a, b, c, and d). Endothelial cell markers (CD34 and CD31) were also positive in necrotic “ghost” cells. Focally, some cells were α-smooth muscle actin positive, which indicates presence of pericytes in cell proliferates (Fig. 3e). More than 10 % of spindled cells were Ki67 positive (Fig. 3f).

Final diagnosis was primary Kaposi’s sarcoma of the heart. The patient had no history of other malignancies and no other primary tumor was detected on CT or Magnetic Resonance Imaging (MRI). Patient was tested negative for HIV infection.

## Discussion

Our paper presents a case of primary Kaposi’s sarcoma of the heart in non-immunodeficient female patient (Table 1). Clinical presentation of pericardial effusion and cardiac tamponade was not indicative of malignancy. CT coronary angiography and TEE also failed to clarify disease entity. Such lesions may mimic vegetations and thrombi by their appearance and behavior.

In the available literature, we found 10 confirmed cases of primary Kaposi’s sarcoma of the heart. Patients’ age varied between 14 to 60 years with mean age of 41.9. 70 % of patients were more than 38 years old. Most tumors were localized in the right atrium [4–8], with two cases (including ours) of right atrial and pericardial involvement [10], one tumor was located in the left atrium [9] and one author described multiple lesions of the heart and pericardium [11]. In most cases tumor arose in the auricle of the right atrium. In these cases disease was diagnosed either postmortem (due to cardiac rupture) or during imaging procedure due to heart failure. Clinical presentation was almost similar in all cases.

Rarity of primary KS of the heart is not the only problem during histological evaluation of the tumor specimens. These lesions contain many necrotic areas, hemorrhages and thrombi, which complicates the tumor sampling. It is very important to perform a careful gross pathological examination and dissection (in case of postmortem study) or tumor tissue sampling (in case of surgical specimen). Pathologists have to promptly examine all suspicious tissues for histology. As shown by others [11] and in our case, main pathognomonic lesions, which give a clue for differential diagnosis, are located at the borders of the lesions. In addition, there are other malignant vascular tumors (spindle cell hemangioma (SCH), angiosarcoma with predominant spindle cell morphology and Kaposhiform haemangioendothelioma) which clinically and histologically mimic Kaposi’s sarcoma. As shown by other authors [22, 23] immunohistochemistry cannot clarify the diagnosis, because the molecular features of these tumors overlap. Although the authors noted [23], that the reactivity of CD34 is stronger in cases of skin Kaposi’s sarcoma in comparison with a weak and focal reaction in angiosarcomas, in individual cases, like the rare tumors of the heart, the immunohistochemical investigation cannot discriminate angiosarcoma from Kaposi’s sarcoma. This method only confirms vascular, endothelial origin of tumor cells and their proliferative capacity. In these cases, differential diagnosis basically lay on histological and cytological features of the tumor. Unlike our case, spindle cell hemangioma contains distinctive epithelioid cells containing vacuoles or intracytoplasmic lumens. The spindled areas of the SCH contain collapsed vessels, pericytes, and fibroblastic cells - all the elements of the vessel wall. Kaposhiform haemangioendothelioma was excluded base on clinical and histopathologic data. It’s occurs nearly exclusively during the childhood. In addition, our case didn’t contain areas with glomeruloid nests, epithelioid endothelial cells and hyaline globules, which are distinctive feature of the kaposhiform haemangioendothelioma. More poorly differentiated vascular tumor, such as angiosarcoma with predominant spindle cell morphology, exhibits more pleomorphism, and contains higher number mitotic cells, than Kaposi’s sarcoma.

In some cases, exact type of tumor has no great impact on clinical outcome (lethal heart tamponade, postmortem diagnosis) but identification of the correct histologic type of the surgically resected tumor may be meaningful for consideration of adjuvant chemotherapy [22].

## Conclusion

In summary, we report a rare case of primary KS of the heart, which showed overlapping clinical, radiological, histopathological and molecular features with other neoplastic and non-neoplastic lesions. Only histological and cytological features gave a clue about nature of the lesion. Immunohistochemistry in such cases plays a role of ancillary study and confirms the endothelial origin of tumor cells. It is helpful in cases when tumor mostly consists of diffuse proliferation areas mimicking fibrosarcomas [21, 20]. We showed that adequate sampling for cardiac tumors has a great importance, because they contain large areas of necrosis, hemorrhage and thrombi complicating the precise recognition of tumorous tissues.

## Consent

Written informed consent was obtained from the patient for publication of this Case Report and any accompanying images. A copy of the written consent is available for review by the Editor-in-Chief of this journal.

## Additional files

Additional file 1:
**3D Computer Tomography.**
Additional file 2:
**Transesophageal echocardiography.**
Additional file 3:
**Transoesophageal Echo-Doppler.**

## Abbreviations

KS: Kaposi’s sarcoma
CT: Computed Tomography
TEE: Transesophageal echocardiography
HHV8: Human herpesvirus 8
αSMA: Alpha-Smooth Muscle Actin
MRI: Magnetic Resonance Imaging
SCH: Spindle cell hemangioma
